# The Anti‐Lupus Plate: Mapping Nutritional Interventions to Inflammatory Pathways in Systemic Lupus Erythematosus

**DOI:** 10.1002/fsn3.70890

**Published:** 2025-09-01

**Authors:** Xiaowei Zhang, Guoliang Fan

**Affiliations:** ^1^ Department of Rheumatology, the First Affiliated Hospital Inner Mongolia Medical University Hohhot China; ^2^ Department of Physics, School of Physical Science and Technology Inner Mongolia University Hohhot China

**Keywords:** anti‐inflammatory diets, diet‐drug interactions, gut microbiota, nutritional interventions, SLE management, systemic lupus erythematosus

## Abstract

A complicated autoimmune illness known as systemic lupus erythematosus (SLE) is typified by multi‐organ dysfunction and ongoing inflammatory processes. Although medication is still the mainstay of managing SLE, tailored nutrition provides a mechanistically sound way to reduce inflammation and promote immunological balance. The present article links certain nutritional factors to pathways controlling cytokine equilibrium, oxidative damage, endothelium integrity, and regulatory T cell activity. These elements include unsaturated fats, vitamins, polyphenols, probiotics, and fiber‐derived short‐chain fatty acids (SCFAs). Additionally, it assesses microbiome‐based therapies and looks at nutritional habits that may have anti‐inflammatory properties, such as the Mediterranean style of eating. Based on these findings, we suggest an evidence‐based “anti‐lupus plate” approach that takes into consideration renal function, concurrent medical conditions, and drug–nutrient relationships while matching dietary habits to pathophysiological goals. Research limitations are highlighted as major obstacles, especially the lack of broad, biomarker‐driven research. These shortfalls must be filled in order to integrate food into a research‐based SLE treatment. Regulated procedures, extended follow‐up, and multimodal dietary treatments are necessary to achieve this.

## Graphical abstract

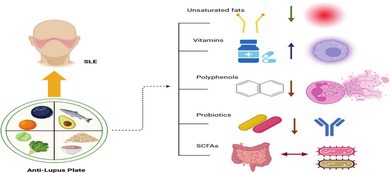

## 1 Introduction

**FIGURE 1:**
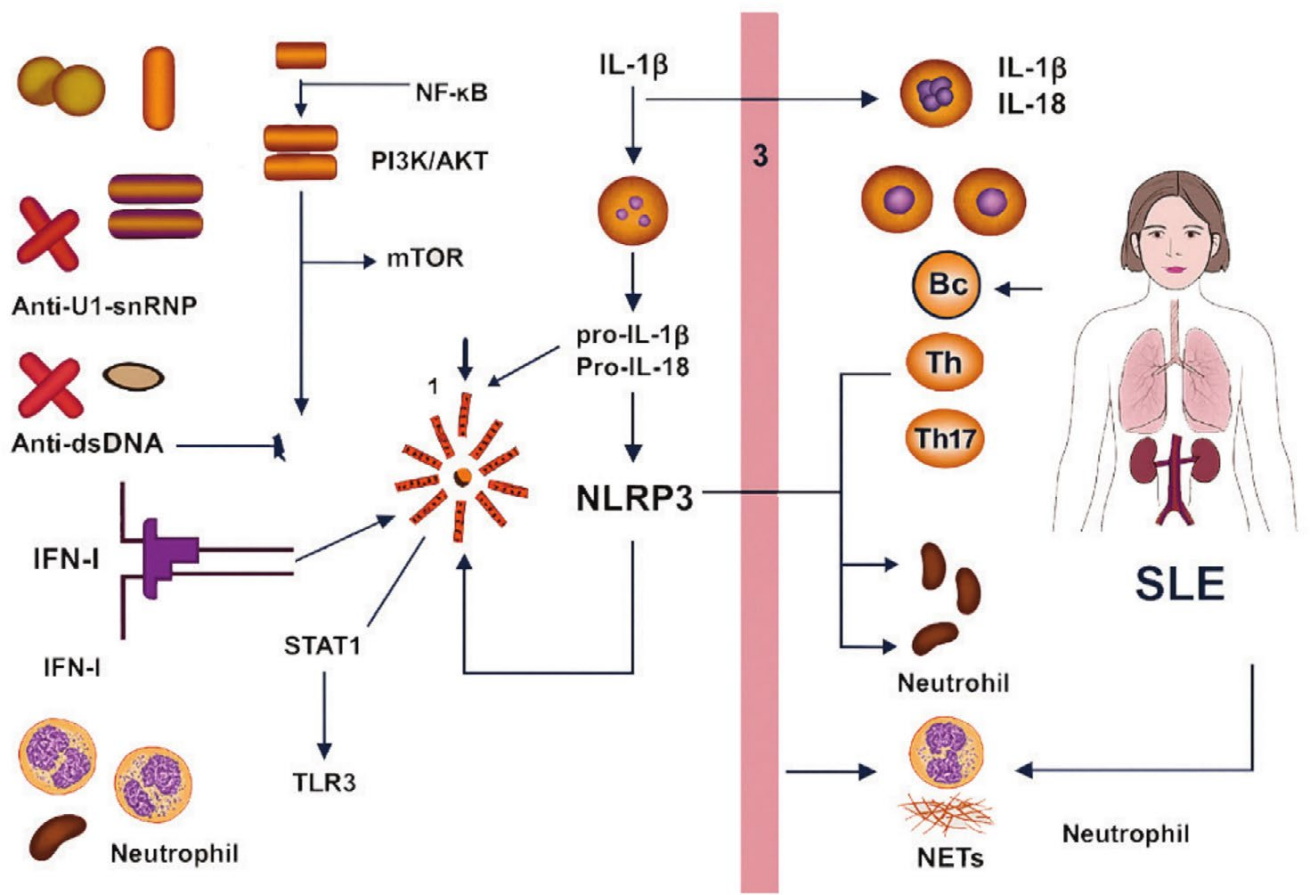
Systemic lupus erythematosus.

**FIGURE 2:**
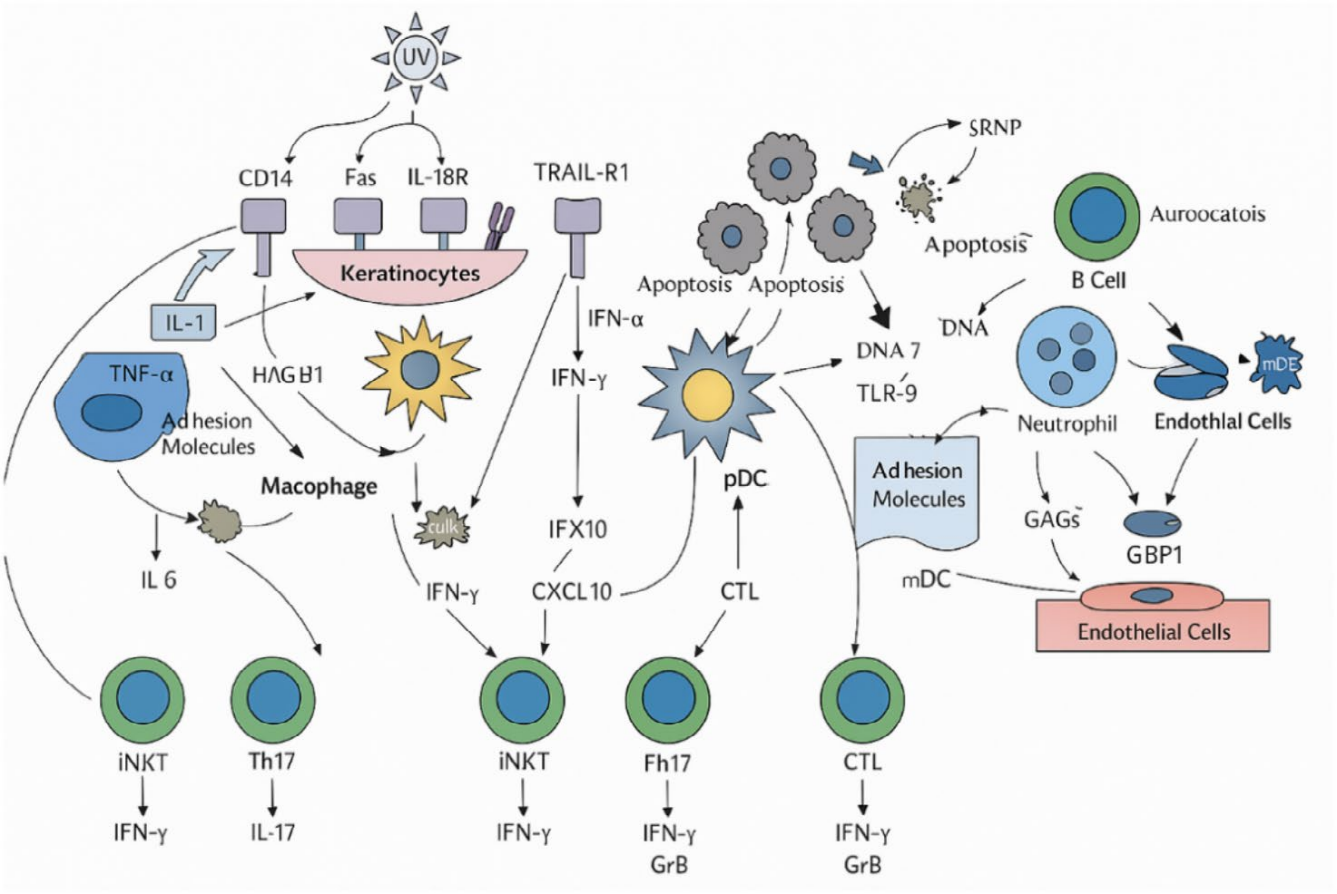
Dermatological lupus erythematosus pathophysiology.

**FIGURE 3:**
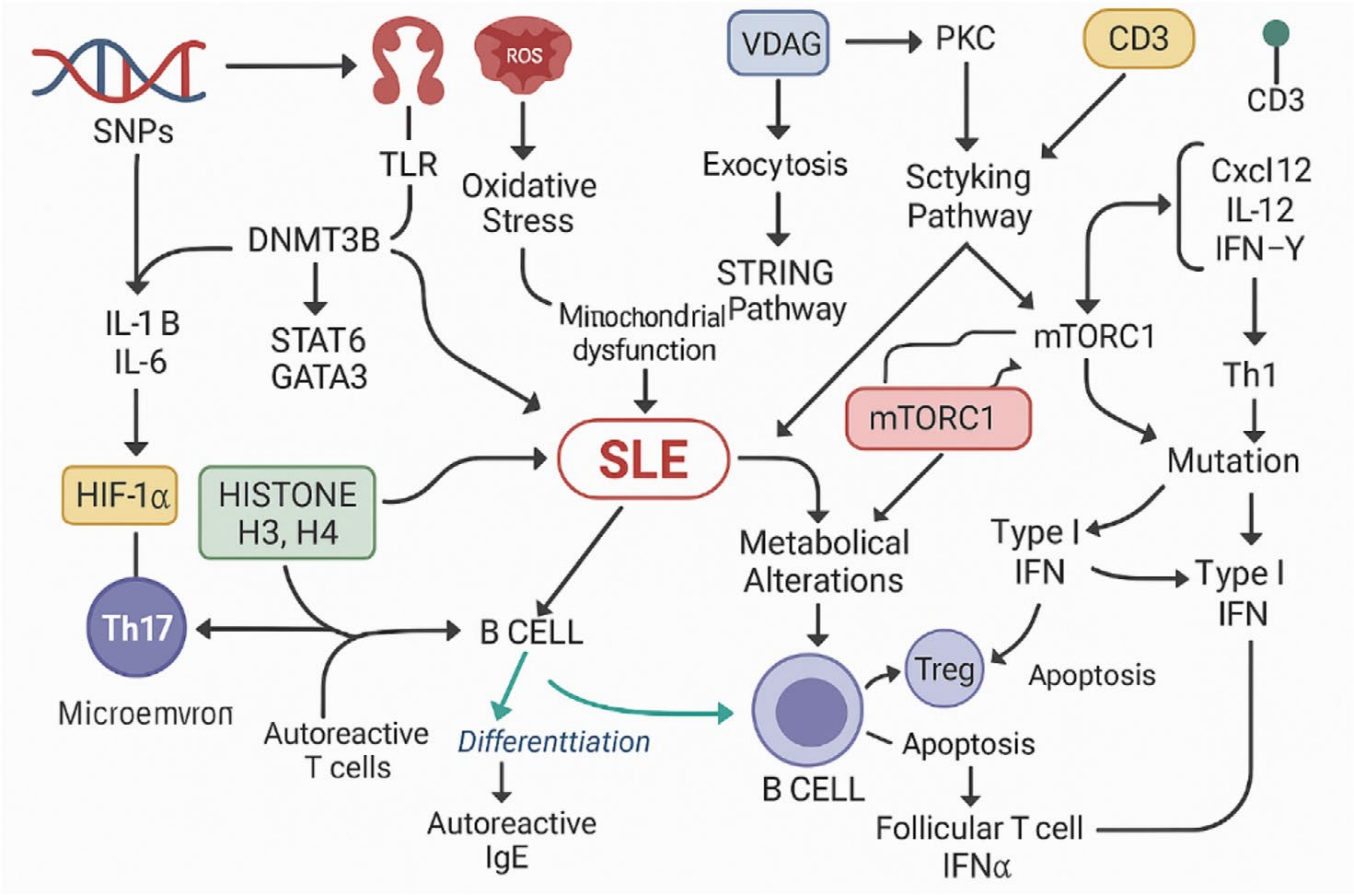
Lupus erythematosus multisystem inflammation and autoantibody production.

**FIGURE 4:**
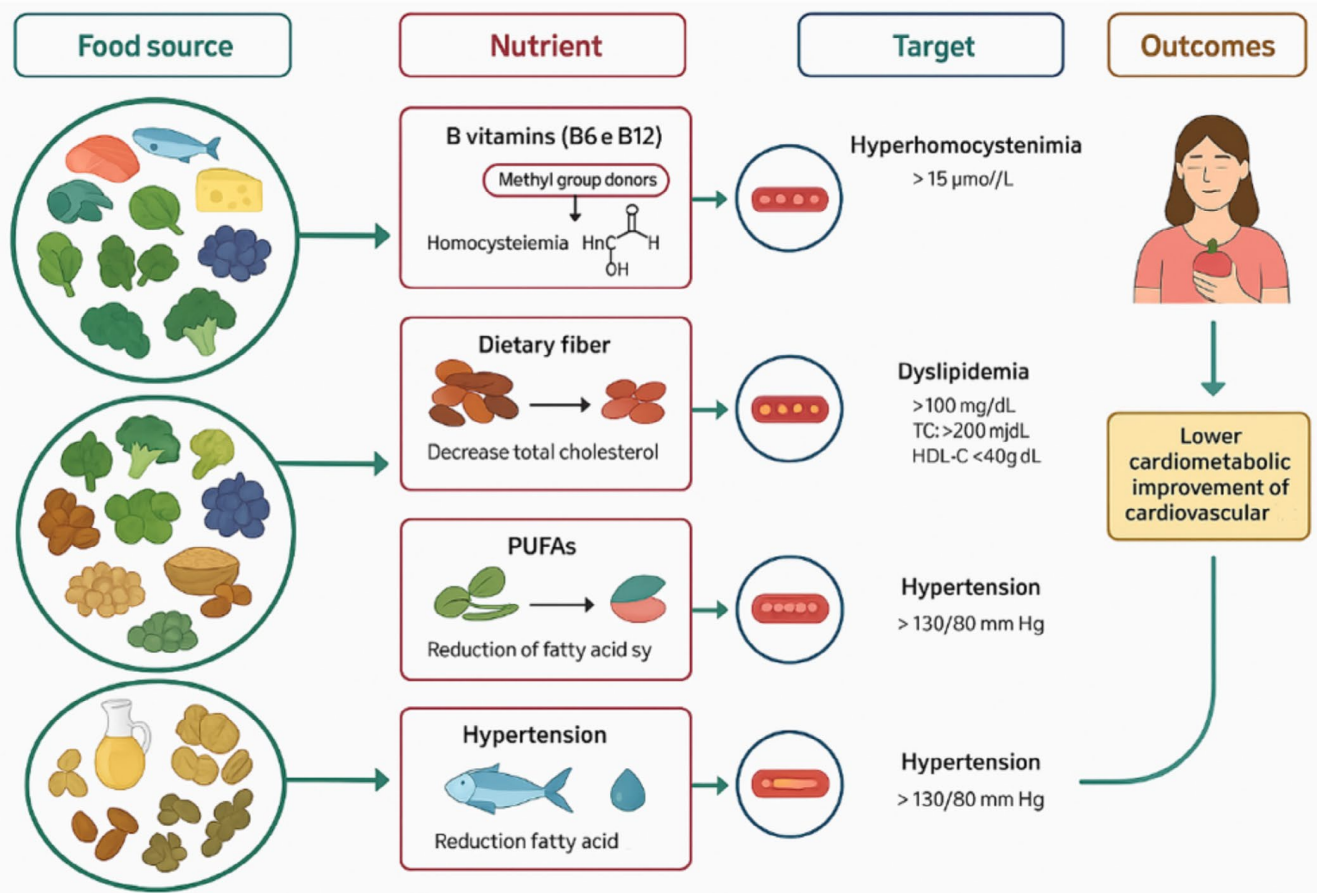
Dietary interventions in SLE.

Systemic lupus erythematosus (SLE) is an autoimmune disease characterized by abnormal immune functions and generalized inflammation that may involve various organs in the body, such as the skin, joints, kidneys, brain, and cardiovascular system. Cell‐wise, SLE is characterized by loss of self‐tolerance, over‐activation of B and T lymphocytes, production of pathogenic autoantibodies (especially anti‐nuclear antibodies), and deposition of immune complexes, which synergistically contribute to tissue injury (Barber et al. 2023). SLE is particularly predilect in childbearing‐aged women and is more common in specific ethnic groups such as African American, Hispanic, and Asian communities. Prevalence rates across the globe range from 20 to 150 cases per 100,000 people (Essouma and Noubiap 2024). SLE etiology is multifactorial, with genetic susceptibility, epigenetic alterations, environmental exposures (such as Ultraviolet [UV] radiation, viral infections), and hormonal effects. Importantly, modifiable lifestyle factors, including diet and physical activity, are now recognized as potential contributors to disease onset and progression. The inflammatory load in SLE is fueled by a vicious circle of immune dysregulation. It revolves around an overproduction of pro‐inflammatory cytokines including interleukin‐6 (IL‐6), tumor necrosis factor‐alpha (TNF‐α), and interferon‐alpha (IFN‐α), all of which enhance immune cell activation and underpin the clinical features of the disease (Jawale et al. 2025). They also initiate downstream pathways like the nuclear factor‐κB (NF‐κB) and janus kinase/signal transducer and activator of transcription (JAK/STAT) signaling cascades, enhancing further inflammation and oxidative stress. Oxidative stress, as indicated by the disproportion between reactive oxygen species (ROS) and antioxidant defenses, accelerates cellular damage and sustains autoimmune responses (Wang, Sun, et al. 2024; Wang, Wang, et al. 2024). Also, gut microbiota dysbiosis, a process called dysbiosis, has more and more been implicated in immune dysfunction in SLE, emphasizing again the intricate nature of the inflammatory picture (Ali et al. 2025). Even with pharmacological developments, existing therapies such as corticosteroids, antimalarials, and immunosuppressants do not cure the disease and can have side effects like metabolic imbalances, osteoporosis, and cardiovascular involvement. These constraints emphasize the pressing need for adjunctive measures that are capable of safely modifying inflammation and enhancing patient outcome. Increasing evidence suggests that diet could play a pivotal role in regulating inflammation and immune responses in chronic disease, including autoimmune disease. In SLE, dietary factors can affect critical molecular pathways contributing to disease pathogenesis. For example, omega‐3 polyunsaturated fatty acids (PUFAs) have been demonstrated to inhibit NF‐κB signaling and suppress the production of pro‐inflammatory cytokines (Tantipaiboonwong et al. 2021). Similarly, foods rich in antioxidants can counteract oxidative stress, and certain dietary habits—such as the Mediterranean diet (MD)—have been linked to lower disease activity in SLE patients (Gavilán‐Carrera et al. 2024). This article attempts to comprehensively lay out dietary interventions over the different inflammatory and immunologic mechanisms contributing to SLE. Rather than advocating general dietary advice, this review synthesizes cutting‐edge evidence on nutritional immunology with SLE's characteristic pathophysiology, documenting how diet may influence: Cytokine networks and inflammatory mediators, oxidative stress and mitochondrial dysfunction, autoantibody generation and lymphocyte activation, gut microbiota composition and gut‐derived inflammation. In the process, an aim is to present a science‐based but readable resource for patients, nutritionists, and clinicians. The following sections of this review will discuss each of these targets in turn, assessing existing evidence for specific nutrients (e.g., flavonoids, omega‐3s, vitamins), eating patterns (e.g., Mediterranean, anti‐inflammatory diets), and functional foods (e.g., cruciferous vegetables, fermented foods) with the ability to influence the SLE inflammatory environment. This review lends proof in favor of integrating nutritional therapy into routine SLE management, not just relieving symptoms, but ultimately promoting long‐term health and remission from disease (Figures 1, 2, 3, 4).

## 2 Pathophysiology of SLE: A Focus on Inflammatory Pathways

### 2.1 Immune Dysregulation in SLE Overview

Decline of self‐tolerance in SLE arises because autoreactive B cells and T cells are evading regulatory surveillance, which leads to chronic inflammation and tissue damage (Pisetsky 2023). B cell hyperactivation generates high titers of autoantibodies, such as anti‐nuclear antibodies (ANA) and anti‐double‐stranded DNA (dsDNA) promoted by T helper cells, including T helper 17 (Th17) and T follicular helper cells (Tfh) that cause germinal center (GC) formation. Inflammation is upheld by a decreased elimination of immune‐mediated complexes and dying cells, which triggers the production of IFN‐α by plasmacytoid dendritic cells (pDCs) (Xiang et al. 2023). Regulatory T cells (Tregs) frequently exhibit malfunctions that allow for unbridled autoreactivity. Toll‐like receptor (TLR) stimulation and neutrophil extracellular trap (NET) development are two intrinsic immune pathways that exacerbate tissue injury and swelling (Winchester et al. 2021).

### 2.2 Cytokines and Signaling Pathways in SLE

SLE is largely caused by type I interferons, particularly IFN‐α. Employing toll‐like receptor 7/9 (TLR7/9), pDCs detect immunological structures comprising genetic material and produce IFN‐α, which promotes the distribution of antigens, B cell stimulation, and interferon‐activated gene expression, also known as an “interferon signature” that is connected to illness progression (Ngo et al. 2024). TNF‐α, which is capable of pro‐ and anti‐inflammatory activities, is increased in lupus nephritis (LN) and activates NF‐κB by signaling through TNFR1/2, which causes proinflammatory enzymes, attachment molecules, and chemical messengers (Winchester et al. 2021). According to Wang, Sun, et al. (2024) and Wang, Wang, et al. (2024), eruptions are associated with greater amounts of IL‐6, which encourages Th17/Treg asymmetry, plasma cell differentiation, and B growth. In order to exacerbate inflammation in the vessels, Th17 cells' interleukin‐17 (IL‐17) attracts neutrophils and combines with IFN‐γ and TNF‐α. NF‐κB maintains a harmful cycle that promotes autoimmunity and is continuously triggered by TLRs, TNF‐α, and ROS (Huang et al. 2022).

### 2.3 Oxidative Stress and Mitochondrial Dysfunction

Oxidative damage from too many reactive oxygen compounds and weakened protection against antioxidants are characteristics of SLE (Perl 2013). ROS increase susceptibility by altering autoantigens and causing harm to parts of cells. T cells with malfunctioning mitochondria generate too much ROS and secrete mitochondrial DNA (mtDNA), which triggers the cyclic GMP–AMP synthase–stimulator of interferon genes (cGAS–STING) and toll‐like receptor (TLR9) networks to discharge type I interferons (Tao et al. 2024). Decreased adenosine triphosphate (ATP) levels, mitochondrial excessive polarization, and decreased antioxidants such as glutathione (GSH), vitamin E, and selenium all contribute to immunological impairment (Sharabi and Tsokos 2020).

### 2.4 The Gut–Immune Axis and Intestinal Permeability in SLE

In SLE, microbial diversity is usually characterized by altered abundances of certain bacteria like Lactobacillus, Ruminococcus, and Bacteroides (Ali et al. 2025), which disrupts intestinal barrier integrity, enhancing gut permeability (“leaky gut”), permitting translocation of microbial products such as lipopolysaccharide (LPS) into the bloodstream, which stimulates toll‐like receptor (TLR4) and causes systemic inflammation. Additionally, reduction of anti‐inflammatory SCFAs decreases Tregs protection and epithelial barrier function (Kim 2023). Increased gut permeability has been associated with increased immune activation and disease activity in lupus‐prone mice and human SLE populations. Interventions aimed at maintaining gut barrier function and microbiota homeostasis, including prebiotics/probiotics supplementation and dietary modifications, hold promise in decreasing inflammation and autoimmune response (Manfredo Vieira et al. 2018).

### 2.5 Anti‐Inflammatory Diets and SLE: Theoretical and Clinical Foundations

#### 2.5.1 Mediterranean Diet (MD)

MD, as per the historical dietary habits of Mediterranean coastal states, is characterized by low consumption of processed food and red meat and high consumption of fruits, vegetables, whole grains, legumes, nuts, seeds, and olive oil, and also fish and chicken. Its anti‐inflammatory properties are contributed to by increased consumption of monounsaturated fats (olive oil), polyunsaturated omega‐3 fatty acids (fish), antioxidants, polyphenols, and dietary fiber (Gavilán‐Carrera et al. 2024). This diet inhibits NF‐κB, prevents the release of pro‐inflammatory cytokines (e.g., IL‐6, TNF‐α), and lowers oxidative stress, thereby possibly regulating autoimmune pathways (Castro‐Barquero et al. 2020). Evidence in autoimmune disorders, such as rheumatoid arthritis (RA), has been linked to reductions in disease severity, enhanced joint function, and lower C‐reactive protein (CRP) (Barbalace et al. 2025). A cross‐sectional study by (Gavilán‐Carrera et al. 2024) revealed that female SLE patients with greater adherence to the MD presented lower disease activity Systemic Lupus Erythematosus Disease Activity Index (SLEDAI) scores, less fatigue, and better metabolic profiles. The fiber and polyphenol‐rich food intake presumably favors the gut microbiota and the immune system, relevant in SLE pathophysiology.

#### 2.5.2 Dietary Approaches to Stop Hypertension (DASH) Diet

The DASH diet emphasizes vegetables, fruits, low‐fat dairy foods, whole grains, lean meat, and nuts, with restrictions on sodium, red meat, and sugar. It is rich in potassium, calcium, magnesium, fiber, and antioxidants, all of which are beneficial for cardiovascular health and immune modulation (Alwarith et al. 2019). The vascular advantages and reduced salt concentration of the DASH meal may support SLE patients with endothelial abnormalities, which are a prominent factor of cardiovascular vulnerability (Han et al. 2025). Although SLE‐specific evidence is limited, its advantage in related autoimmune and inflammatory diseases is becoming more and more accepted. A paper by (Mangels and Mohler III 2017) demonstrated that DASH diet consumption decreased CRP and TNF‐α levels in patients with hypertension. Increased DASH adherence was correlated with improved lipid profiles and reduced arterial stiffness in SLE groups (Alwarith et al. 2019). The antioxidant and low‐sodium nature of the diet forms the basis for its application in hypertensive or otherwise at higher cardiovascular risk SLE patients.

### 2.6 Nutritional Vulnerability in SLE Patients

In addition to following anti‐inflammatory eating habits, individuals with SLE frequently suffer from inadequate nourishment that could make their condition worse. These risks are caused by a number of variables, such as a lack of renal function, abnormal food metabolism, inadequate absorption, and the adverse reactions of medications (such as immune‐suppressive and corticosteroids) (Kim 2023). Typical inadequacies consist of: Vitamin D deficiency is most common in SLE and occurs due to sun avoidance, renal losses, and inflammation‐driven catabolism. Low vitamin D levels are associated with higher disease activity, fatigue, and greater cardiovascular complications, partly via reduced differentiation of Tregs and increased T helper 1 (Th1) and Th17 activities (Barbalace et al. 2025). The information, however, is not all uniform. Although some research, such as that conducted by Kamen et al. (2006), suggests that inadequate levels of vitamin D could represent a contributory factor for SLE and that vitamin D repletion may improve immunological function, other research contradicts this. Likewise, antioxidants like vitamin E and selenium play a vital role in preventing oxidative stress and defense against lipid peroxidation and immune dysfunction. Their deficiencies can exacerbate ROS‐mediated tissue injury in SLE (Aparicio‐Soto, Sánchez‐Hidalgo, and Alarcón‐de‐la‐Lastra 2017; Aparicio‐Soto, Sánchez‐Hidalgo, Cárdeno, et al. 2017). Moreover, omega‐3 PUFAs are also commonly lacking in SLE patients, adding to the pro‐inflammatory condition. Protein‐energy malnutrition and sarcopenia occur in active SLE, especially in patients with renal or gastrointestinal disease. This compromises immune function, healing of wounds, and infection risk. In addition, corticosteroids and immunosuppressives can cause hyperglycemia, dyslipidemia, and alterations in body composition, further worsening the nutritional status (Aparicio‐Soto, Sánchez‐Hidalgo, and Alarcón‐de‐la‐Lastra 2017; Aparicio‐Soto, Sánchez‐Hidalgo, Cárdeno, et al. 2017). Nutritional management of deficiencies plays a central role in the treatment of SLE. Nutritional evaluation and management according to individual requirements can optimize treatment effect, enhance quality of life, and decrease the frequency of flares (Tables 1, 2, 3).

**TABLE 1:** SLP‐related treatment and outcomes.

SLP treatment	Rationale	Outcome	References
Omega‐3 fatty acids supplementation	Anti‐inflammatory effects through modulation of cytokine production	Reduced inflammation; improved clinical markers in SLE	Calder (2015)
Mediterranean diet	Reduces oxidative stress and systemic inflammation	Improved arterial stiffness and decreased medication use	Gavilán‐Carrera et al. (2024)
Fecal Microbiota Transplantation (FMT)	Restores gut microbiome balance to modulate immune response	Preliminary decrease in SLE activity	Huang et al. (2022)
Diet‐Gut Microbiota Modulation	Diet influences gut microbiota diversity, impacting immune regulation	Positive association with reduced autoimmunity	Vieira et al. (2014)
Omega‐3 supplementation	Targets pro‐inflammatory cytokine pathways	Clinical improvements in SLE patients	Ramessar et al. (2022)
Probiotics/Synbiotics	Enhance immune regulation and gut health	Improved immune function and clinical symptoms	Askari et al. (2021)
Vitamin D supplementation	Deficiency linked to SLE activity; Vitamin D modulates immune response	Reduced fatigue and disease activity in adolescents	Lima et al. (2016)
Antioxidant therapies	Oxidative stress contributes to SLE pathogenesis	Potential therapeutic benefits via reduction of oxidative stress	Perl (2013)
Low glycemic index diet	Controls weight and inflammation in corticosteroid‐treated patients	Improved fatigue and weight management	Davies et al. (2012)
Low‐fat vegan diet	Reduces systemic inflammation	Improved symptoms in autoimmune disease patients	McDougall et al. (2002)
ω‐3 polyunsaturated fatty acids	Improve endothelial function; reduce inflammation	Positive effect on disease activity and vascular health	Wright et al. (2008)
Vitamin D therapy	Supports immune modulation	Reduction in disease flares and improved immune balance	Mak (2018)
Diet and nutrients	Comprehensive review of diet's immunomodulatory effects	Identified several beneficial dietary patterns and nutrients	Islam et al. (2020)
Antioxidant‐enriched diet	Targets oxidative stress and inflammation gene expression	Downregulation of inflammatory pathways	Gualtieri et al. (2023)
Genetic‐based omega‐3 personalization	Individual genetic variants affect omega‐3 metabolism	Suggests need for personalized nutrition approaches in SLE management	Loukil et al. (2024)

**TABLE 2:** Drug‐nutrient interactions and clinical implications.

Medication	Nutrient (s)	Mechanism	Implications	References
Glucocorticoids (prednisone)	Calcium, vitamin D	By inhibiting calcium carriers and vitamin D channel signaling, glucocorticoids decrease intestinal calcium uptake. They also enhance renal calcium elimination and effectively prevent osteoblast growth and longevity while extending osteoclast lifespan	Greater likelihood of fragility fractures and a higher prevalence of osteopenia/osteoporosis	Gado et al. (2022)
Methotrexate (MTX)	Folate	Low quantities of methotrexate influence DNA formation in swiftly proliferating cells, by inhibiting dihydrofolate reductase, which lowers folate bioavailability	Enhanced nausea, megalocytosis, and mouth ulcers	Friedman and Cronstein (2019)
Mycophenolate mofetil (MMF)	Fluid/electrolytes, iron, vitamin B9, vitamin B12	Gastrointestinal contamination from MMF is prevalent and includes diarrhea, enteritis, and more rarely, colitis. Severe or protracted diarrhea linked to MMF causes electrolyte and fluid loss in the intestines and may cause inadequate absorption of nutrients	Long‐lasting signs of iron‐insufficiency anemia, hypokalemia, dehydration, or folate deficiencies	Bhattacharya et al. (2022)
Calcineurin inhibitors (tacrolimus, cyclosporine, voclosporin)	Magnesium, potassium	CNIs damage tubules and decrease kidney blood circulation, which can alter potassium and pH equilibrium and result in magnesium elimination via TRPM6/7 modifications	Electrolyte imbalances and hypomagnesemia can happen, contingent upon kidney function and concurrent drugs taken at the same time	Yarandi and Shirali (2023)

**TABLE 3:** Gut microbiota composition of SLE patients.

Analysis	Gut microbiota	Participants	Subgroup/stratified analysis	References
Metagenomic analysis	Reduced Firmicutes/Bacteroidetes Ratio	Adult males and females	N/A	López et al. (2016)
Whole‐genome profiling	The gut microbiota of SLE patients had higher levels of Clostridium species ATCC BAA‐442, *Atopobium rimae* , *Shuttleworthia satelles* , *Actinomyces massiliensis* , *Bacteroides fragilis* , and *Clostridium leptum* . The results obtained from mice and patients were in agreement	SLE patients and mice	N/A	Chen et al. (2021)
16S ribosomal RNA (16S rRNA) gene sequencing	Whilst the genera Dialister and Pseudobutyrivibrio were severely decreased in individuals with SLE, the genera Rhodococcus, Eggerthella, Klebsiella, Prevotella, Eubacterium, Flavonifractor, and Incertae sedis appeared substantially increased	Adult females	N/A	He et al. (2016)
16S rRNA amplicon sequencing and microbiome profiling	The diversity and richness of gut microorganisms were considerably reduced in SLE patients. With fewer species like Ruminococcus 2 and Agathobacter and more Escherichia‐Shigella and Bacteroides, a decreased Firmicutes‐to‐Bacteroidetes ratio was noted. A greater occurrence of Prevotella was linked to a more severe disease	Adult females aged between 18 and 40	*Disease activity*: Increased Prevotella in severe illness. *Medication use*: Reduced Romboutsia with azathioprine; elevated Prevotella with cyclophosphamide. Prednisolone usage > 10 mg/day linked with elevated Romboutsia, Comamonas and Slackia	Ali et al. (2025)
Next‐generation sequencing (NGS)	Microbiota diversity and richness were lower in TC (SLE) mice than in C57/B6 mice	Female mice	N/A	Ma et al. (2019)
16S rRNA gene sequencing	Patients with SLE showed a decreased F/B ratio and an overall decline in alpha‐diversity	Females age ≥ 18 years Female mice	Disease activity: Unsupervised groupings of individuals with SLE indicated a subgroup with more altered microbial diversity, which was significantly associated with an elevated SLEDAI rating	Toumi et al. (2022)

Epidemiological evidence has repeatedly correlated diet quality with inflammation and autoimmune disease risk. In a large sample prospective cohort study, increased compliance with anti‐inflammatory eating habits reduced autoimmune disease risk, including SLE (Gavilán‐Carrera et al. 2024). In patients with SLE, in research utilizing dietary quality indexes like Healthy Eating Index or MD Score, reverse correlations with SLEDAI, CRP, and fatigue have been seen (Tümkaya Yılmaz et al. 2022). A 2021 systematic review concluded that healthy eating patterns, especially with high consumption of plant foods and omega‐3 fatty acids, were associated with improved clinical outcomes in autoimmune disease, such as lupus. While there are fewer RCTs of SLE compared to RA or MS, there are some encouraging interventional data. Research showed that fish oil supplementation in SLE patients decreases disease activity and enhances endothelial function (Ramessar et al. 2022). In a randomized controlled trial of RA patients, compliance with a MD was associated with lower joint pain, morning stiffness, and enhanced physical function (Sharabi and Tsokos 2020). Vegan diets heightened inflammation and pain indices in RA. There are fewer comparable trials in SLE, but similar benefits are extrapolated based on similar inflammatory mechanisms. Newer evidence indicates that dietary interventions for the reconstitution of gut microbiota homeostasis (e.g., high‐fiber diet) are potentially useful to SLE patients (Ali et al. 2025). Despite such promising findings, SLE‐specialized trials comparing full‐diet interventions are lacking, highlighting a call for more studies.

## 3 Dietary Modulation of Inflammatory Pathways in SLE

In order to manage SLE, nutritional therapy—which includes dietary changes and the use of nutritional supplements—may be a viable strategy. By lowering co‐morbidities and enhancing patients' quality of life, it may help avoid flare‐ups and minimize side effects in comparison to traditional pharmaceutical therapy (Islam et al. 2020). The course of SLE may therefore be influenced by the dietary habits and nutritional condition of individuals. Moderate protein and energy intake combined with a diet high in vitamins, minerals, and mono‐ and polyunsaturated fatty acids—particularly antioxidants—can have a positive protective impact against tissue damage and reduce inflammation (Jiao et al. 2022).

### 3.1 Macronutrients and Their Role in Inflammatory Modulation

#### 3.1.1 Fiber

Dietary fiber is composed of edible polymers of carbohydrates with three or more monomeric units that are not digested or absorbed in the small intestine given that they are resistant to endogenous digestive enzymes. Because dietary fiber helps lower serum levels of inflammation indicators such as homocysteine, cytokines, and CRP, it is advised that people with SLE consume plenty of it. Low fiber intake is a dietary trait observed in SLE patients that seems to be linked to worsening activity of the illness (Pocovi‐Gerardino et al. 2018; Elkan et al. 2012). According to a prospective study of female Japanese patients, eating enough fiber lowers the incidence of SLE (Minami et al. 2011). Dietary fiber deficiency increases the pathophysiology of lupus and the corresponding immunological dysregulation (Schäfer et al. 2021). Because dietary fiber is less readily available, low levels of SCFAs, a byproduct of fiber fermentation, can lead to inflammation and a disorder in both innate and adaptive immunity.

#### 3.1.2 Fats

Unsaturated fats appear to have a beneficial influence on regulating the inflammatory process, whereas saturated fats have a detrimental effect on the progression of disease, most likely because they promote inflammation. PUFAs (omega‐3 and omega‐6) are vital nutrients that the body is unable to produce on its own (Coniglio et al. 2023). They are necessary for controlling cell signaling, preserving the fluidity of cell membranes, and inhibiting inflammatory processes. These roles suggest that PUFAs may influence the pathophysiology of SLE by altering the immune regulatory system, lowering the synthesis of inflammatory mediators, and modifying immune cell activity. Alpha‐linolenic acid, docosahexaenoic acid, and eicosapentaenoic acid are examples of omega‐3 fatty acids, which are PUFAs (Mariamenatu and Abdu 2020). In general, omega‐3 fatty acids possess anti‐inflammatory qualities and help lower disease activity in a number of inflammation‐related diseases, including SLE. Compared to controls, SLE patients have a distinct macronutrient dietary pattern, and it has been noted that they consume less omega‐3 fatty acids (Elkan et al. 2012). Regardless of their prior history of cardiovascular disease, SLE patients exhibit changed plasma and fatty acid composition that promotes inflammation (Aghdassi et al. 2011). According to research, omega‐3 fatty acids lower the levels of chemokines, eicosanoids, CRP, cytokines, and other inflammatory mediators. According to a randomized clinical trial, taking omega‐3 fatty acid supplements significantly reduced the level of CRP (Curado Borges et al. 2017). According to another randomized trial, SLE patients who take low‐dose dietary supplements of omega‐3 fish oils may help their cardiovascular health in addition to improving endothelial function and lowering oxidative stress (Wright et al. 2008). According to a double blind, double placebo controlled factorial trial, fish oil (omega 3) supplements may help manage SLE (Duffy et al. 2004). A population based cohort study revealed that there is a positive correlation between patient‐reported outcomes in SLE and higher dietary consumption of omega‐3 fatty acids (Charoenwoodhipong et al. 2020).

On the other hand, high levels of omega‐6 fatty acids can be pro‐inflammatory, but they are also essential for healthy cellular and immunological responses. According to a cohort study by Charoenwoodhipong et al. (2020), lower *n*−6:*n*−3 ratios are positively correlated with patient‐reported outcomes in SLE, namely self‐reported lupus activity and sleep quality. According to studies, consuming more omega‐6 than omega‐3 is linked to an increased risk of chronic disease and inflammation. The precise functions of omega‐6 fatty acids and the ideal omega‐6 ratio in the treatment of SLE must thus be further investigated.

#### 3.1.3 Proteins and Amino Acids

Numerous studies have examined dietary protein restriction in SLE patients and animal models. Because non‐protein calories are redirected, excessive protein consumption may paradoxically result in a loss of muscle mass. This leads to decreased lean mass, which is closely associated with poor bone mineral density, a complex problem seen in JSLE and JIA (Kędzia et al. 2023). Research indicates that plant‐based diets, which tend to be lower in protein, can enhance immunological modulation and reduce the generation of pro‐inflammatory cytokines. It has also been hypothesized that certain amino acids, in addition to proteins, may have an impact on how SLE develops. Blood tyrosine levels were shown to be considerably lower at the metabolite level in infants born to moms with SLE (Cai et al. 2024). Researchers discovered that SLE patients had significantly greater amounts of L‐canavanine when compared to healthy people. Treatment for SLE may benefit from taurine. In lupus‐prone mice given a high‐cholesterol diet, taurine improved protective pathways and decreased heart cell death, fibrosis, and damaging signaling, protecting the hearts of the mice (Hsu et al. 2008). According to Huang et al. (2009), these findings imply taurine may help prevent heart issues in mice and possible protective effects of taurine in the management of CVD in SLE. Therefore, limiting the intake of certain amino acids, such as phenylalanine and L‐canavanine, and maintaining a moderate protein intake help SLE patients.

### 3.2 Micronutrients and Bioactive Compounds in SLE

#### 3.2.1 Vitamins

Numerous epidemiological investigations have looked into the possible impact of antioxidant food intake and supplementation in SLE patients. In SLE, oxidative stress serves as a trigger for autoimmunity, causing aberrant apoptotic processes, immune system dysfunction, and the generation of autoantibodies (Perl 2013). Suppressing oxidative stress pathways may thereby lessen the toxicity of immunosuppressive treatments, lower disease activity, and enhance the quality of life for SLE patients. A vital component of fat‐soluble vitamins, vitamin A serves a variety of purposes, including preserving the integrity and healthy operation of the immune system in addition to influencing nuclear retinoic acid receptors, which control the transcription of numerous genes. In SLE patients, vitamin A can alter the proportion of Th17 and Treg cells. According to a matched case–control study, vitamin A levels are significantly lower in SLE patients, and this has a negative correlation with the Th17 population. Retinoic acid treatment in vitro can also reduce the Th17/Treg ratio, promote Treg differentiation, and suppress Th17 differentiation in SLE patients (Handono et al. 2016). According to a hospital‐based case–control study, Th17/Treg balance is impacted by vitamin A deficiency, which is a poor prognostic factor in SLE patients. Regular retinoic acid administration may improve the prognosis of SLE patients and be a promising supplemental treatment (Fettouh et al. 2019).

Vitamin D reduces Th1 cell growth, increases self‐tolerance, and strengthens the innate immune response. Activated B cell growth is inhibited. The antiproliferative effect on B cells is mediated by the binding of calcitriol (1,25(OH)_2_D_3_) to the vitamin D receptor (VDR), which is expressed by B cells. Vitamin D supplementation with routine monitoring should be taken into consideration as part of their health management plans because a systematic review and meta‐analysis revealed that patients with SLE have significantly higher levels of inadequate serum vitamin D than healthy subjects (Islam et al. 2019). According to a case–control study by Ben‐Zvi et al. (2010), vitamin D administration has been shown to have inhibitory effects on DC maturation and activation, which may help SLE patients regain immunological homeostasis. According to a systematic review and meta‐analysis, individuals with SLE, vitamin D significantly raised C3 levels and significantly decreased SLEDAI scores (Irfan et al. 2022). According to a prospective interventional study, individuals with SLE who were vitamin D deficient, vitamin D plus calcium administration dramatically increased bone mineral density (Al‐Kushi et al. 2018). According to Yazdanpanah et al. (2017), vitamin D3 may have immunomodulatory effects on SLE patients by altering the expression levels of specific TLRs. An increase in T‐reg cells and Th2 cytokine production may be anticipated in SLE patients following a prolonged course of vitamin D treatment (Piantoni et al. 2015). Vitamin C may have a positive impact on inflammation and the repair of aberrant immunological components via mediating the oxidative stress response in SLE. According to a prospective study, consuming vitamin C may help reduce the development of active SLE disease because it is negatively correlated with the risk of active disease (Minami et al. 2003). The traditional CAD risk variables are insufficient to account for the extra morbidity and mortality associated with coronary artery disease (CAD) in patients with SLE. Reduced lipid peroxidation was linked to the combined treatment of vitamins C and E (Tam et al. 2005).

#### 3.2.2 Minerals

Through various immunomodulatory processes, some trace metals, such as zinc (Zn), copper (Cu), and selenium (Se), often reduce SLE activity. When compared to the control group, SLE patients' serum ZNF‐76 levels showed a statistically significant decline. Reduced serum ZNF‐76 mRNA expression is strongly linked to a higher risk of developing SLE (Ahmed et al. 2025). According to a metanalysis based on case–control studies, individuals with SLE had noticeably reduced levels of Zn and Fe. Furthermore, SLE patients have noticeably higher Cu levels (Wang et al. 2023). Trace element and hazardous metal profiles in SLE patients differ from those in healthy controls, according to an animal‐based investigation. Reduced levels of vanadium (V), zinc (Zn), and lead (Pb) and elevated (Lithium) Li were found to be linked to the diagnosis of SLE. An important trace element having anti‐inflammatory and immunological benefits is selenium (Se). B cell and macrophage activation, differentiation, and maturation are inhibited by selenium administration. As a possible therapeutic complement for SLE patients, its particular inhibitory effect on B cell activation and GC B cell differentiation may be investigated (Soni et al. 2019). Low selenium levels were associated with increased “bad” cholesterol (c‐LDL) in almost half of adolescents with jSLE (Aires et al. 2022). Moreover, selenium supplementation may significantly enhance a number of metabolic indicators and clinical symptoms in individuals with lupus, as per a randomized controlled trial (Abrishamkar et al. 2024).

#### 3.2.3 Phytochemicals and Polyphenols

In light of their antioxidant qualities, phenolic compounds—which are found in many plants—are an important component of both human and animal nutrition. These substances are among the most prevalent classes of phytochemicals and have significant physiological and morphological significance in plants (Sun and Shahrajabian 2023). Because of their positive effects on human health, dietary polyphenols have garnered a lot of attention in recent years. Oranges, apples, and red wine are among the foods high in polyphenols that may have a favorable impact on gut bacteria in women with lupus (SLE), according to an age‐matched case–control study (Cuervo et al. 2015). According to a randomized controlled trial, using green tea extracts on a regular basis improves various aspects of quality of life and the activity of SLE disease (Shamekhi et al. 2017). Virgin olive oil (VOO) and its phenol fraction (PF) have been demonstrated to focus on and hamper inflammatory responses in the monocyte–macrophage lineage of mice with pristane‐induced SLE (Aparicio‐Soto et al. 2018). According to another research, mice provided EVOO showed increased expression of the nuclear factor erythroid 2–related factor 2 (Nrf‐2) and heme oxygenase‐1 (HO‐1) proteins, as well as significantly improved JAK/STAT, mitogen‐activated protein kinase (MAPK), and NF‐κB pathway activity. These findings confirmed that EVOO is a useful functional food that can help prevent or treat SLE (Aparicio‐Soto et al. 2015).

In another study, dietary phenols reduced pro‐inflammatory cytokines and renal damage in lupus mice. Supplementing with HTy and HTy‐Ac may be a unique dietary strategy for controlling SLE, according to Aparicio‐Soto, Sánchez‐Hidalgo, and Alarcón‐de‐la‐Lastra (2017) and Aparicio‐Soto, Sánchez‐Hidalgo, Cárdeno, et al. (2017). By controlling Th17 differentiation during the formation of LN and targeting the CXCL signaling pathway, fisetin may be a viable treatment for SLE, according to another study. By lowering anti‐ds DNA and IL‐6 levels, curcumin, a safe and effective adjuvant treatment, reduced autoimmune activity and inflammation in SLE patients (Sedighi et al. 2024). According to Liu, Wang, Wu, et al. (2024) and Liu, Wang, Ying, et al. (2024), apigenin (AP) suppresses the autoantigen‐presenting and stimulatory properties of APCs, which are essential for the activation and proliferation of autoreactive Th1 and Th17 cells as well as B cells in lupus. AP inhibits the STAT3/IL‐17 signaling pathway, which causes cluster of differentiation 8 (CD8^+^) T lymphocytes to undergo apoptosis and decreases their ability to attract macrophages. Accordingly, patients with SLE may benefit therapeutically from a diet high in dietary flavonoids, especially AP (Liu, Wang, Wu, et al. 2024; Liu, Wang, Ying, et al. 2024).

Quercetin effectively prevents neutrophils from forming NET. Quercetin inhibited the development of NET in vitro by lowering radicals and citrullinating neutrophil histone deposition (Liu, Wang, Wu, et al. 2024; Liu, Wang, Ying, et al. 2024). According to Chen et al. (2024), it stimulated neutrophil cell death and autophagy to eliminate stimulated cells in arthritic conditions models and lowered P2X7R to p38 MAPK to NOX2 communication, a significant source of ROS. Even in lupus, these implications might inhibit NETosis. Another study showed that quercetin (QC) can prevent the immunosenescent phenotype and has a substantial inhibitory effect on Tfh cell ontogeny. Because of this, QC may be a useful therapy strategy for SLE (Xiong et al. 2024). Resveratrol (RSV) has a strong anti‐inflammatory effect by inhibiting immune cell overactivation, reducing procytokine levels, and inhibiting the production of autoantibodies. As a result, RSV is a beneficial and potential treatment option for SLE (Huo et al. 2024).

RSV significantly reduces the development of NETs. Through the inhibition of MPO action, the reduction of H_2_O_2_ amounts, and the prevention of neutrophil elastase transfer to nuclei, pretreating human neutrophils with RSV in the laboratory decreased NET release in response to various triggers (PMA, LPS, and IL‐8) (de Mattos et al. 2024).

Additionally, SIRT1 is proposed as an alternate target for RSV, which stimulates this gene that codes for proteins in the management of SLE. Increased SIRT1 modulates NF‐κB and ROS/TRPM2/Ca2+ routes, which suppress the NLRP3 inflammasome and slow the course of LN. Accordingly, SIRT1 might be a viable treatment option for LN alleviation (Tian et al. 2023). The percentage of circulating EPCs in SLE patients was positively connected with caffeine intake; additionally, caffeine in vitro treatment was able to enhance EPC vitality and lifespan by inhibiting apoptosis and promoting autophagy via the A2AR/SIRT3/AMPK pathway (Orefice et al. 2025).

### 3.3 Probiotics, Prebiotics and SLE

The link between microorganisms and autoimmune disorders is well established. Autoimmune diseases can result from changes in the microbiome, or “dysbiosis,” which are impacted by specific genetic backgrounds and environmental circumstances. There is increasing evidence that the overgrowth, or bloom, of commensal gut bacteria or pathobionts and a relative decrease in symbiotic bacteria can change the microbiome‐host relationship, upset the physiological balance, and ultimately cause the gut epithelial barrier to become more permeable, resulting in “leaky gut syndrome” (LGS). In patients who are genetically predisposed, this disorder can cause aberrant immune responses to microbial antigens and increase the risk of developing SLE and LN (Parodi et al. 2025). Probiotics, prebiotics, and synbiotics may help restore a balanced microbiota and lessen the negative effects caused by microbial imbalances during the course of the illness. Gut microbiota‐based treatments may help a number of autoimmune illnesses, including SLE, according to a systematic review and meta‐analysis (Zeng et al. 2024). Evidence from murine models of lupus, probiotics and prebiotics help lessen the severity of lupus by improving Th17/Th1 imbalance, lowering autoantibody synthesis, and inducing Treg cell development (Pan et al. 2021). According to a review research, probiotics and prebiotic treatment may help cure SLE by improving intestinal dysbacteriosis (Zhang et al. 2021).

In SLE mouse models and SLE patients, probiotic therapy has shown significant anti‐inflammatory, regulatory, and antiapoptotic effects in addition to improvements in clinical manifestations (Mirfeizi et al. 2023). In a systematic review primarily based on animal studies, probiotic strains like *Lactobacillus fermentum* CECT5716, *Lactobacillus casei* B255, *Lactobacillus reuteri* DSM 17509, *Lactobacillus plantarum* LP299v, and *Lactobacillus acidophilus* have been shown in another systematic review and meta‐analysis to significantly lower levels of pro‐inflammatory cytokines (TNF‐α, IL‐12, IL‐6, IL‐1β, IL‐17, and IFN‐γ) while boosting anti‐inflammatory IL‐10 and Treg cells. Additionally, probiotics slow down the synthesis of autoantibodies, which lengthens the duration of remission, lowers the frequency of flare‐ups, and delays the course of the disease. Additionally, taking probiotics improves intestinal stability, inhibits pathogen colonization, and reduces gut dysbiosis (Goh et al. 2025).

According to a case‐control study, *L. rhamnosus* and *L. delbrueckii* may help treat SLE patients by modifying the expression of miR‐181a and miR‐155 in those with the condition (Vahidi et al. 2018). Another study showed that in a mouse model of lupus caused by toll‐like receptor (TLR‐7) activation, long‐term administration of the probiotics LC40 or BFM reduced endothelial impairment and hypertension (Sánchez et al. 2021). A randomized controlled trial revealed that the outcomes of SLE patients have also been positively impacted by synbiotics, which are a combination of probiotics and prebiotics. According to the research study's findings, the synbiotics group's SLE Disease Activity Index 2000 score and protein and mRNA levels of IL‐17A were considerably lower following the treatment than at baseline. Synbiotic supplementation thus demonstrates promise as a therapeutic adjuvant for the therapy of SLE (Mirfeizi et al. 2024). According to another randomized controlled trial, the rise in hs‐CRP levels might be inhibited by taking a synbiotic supplement. Synbiotic supplementation may thereby change the makeup and roles of the gut microbiota and lower systemic inflammation and SLE disease activity (Widhani et al. 2021).

The empirical proof is still in its infancy and is frequently based upon small‐scale trials with systematic constraints, even though preliminary research clearly shows the immune‐regulating and anti‐inflammatory properties of several food elements in SLE models. Before these therapies are recommended as standard treatment choices for SLE, they must be confirmed by broad, meticulously designed, multicenter controlled randomized studies.

## 4 Research Gaps and Future Directions

There is considerable heterogeneity in the signs and symptoms, magnitude, and attitude towards therapy for SLE, among various demographic groups and biological roots. Genetic, epigenetic, and ecological variables—such as dietary influences—interact extensively during the course of the disease. Individualized dietary methods are still lacking, even though the importance of diet in managing SLE is becoming more widely acknowledged. Personalized healthcare strategies are also becoming more prevalent in the care of the illness.

Throughout several cohorts, Black/African American, Hispanic/Latino, and certain aboriginal peoples have more adverse results, a greater incidence of LN, and greater incidences of SLE than White individuals; accessibility and socioeconomic variables interact with physiological causation. With an increased likelihood of nephritis and cardiovascular disease, dietary recommendations for these individuals should focus on cardiorenal therapy (e.g., sodium limitations, blood pressure regulation, weight control, and cholesterol optimization). KDIGO 2024 recommends < 2 g/day sodium (< 5 g salt) for chronic kidney disease, which is relevant to the treatment of LN in addition to RAAS hindering and blood pressure medications (Buie et al. 2023; Parodis et al. 2023). Although causation has not been established, new preliminary evidence also links greater compliance to a Mediterranean‐style dietary pattern to decreased disease progression and cardiac risk in SLE; considering its cardiometabolic advantages, this dietary regimen seems logical to underline (Pocovi‐Gerardino et al. 2021).

A large number of adult instances of SLE involve women, who also exhibit sex hormone‐linked immunological variations (such as estrogen dose‐related regulation of intrinsic as well as adaptive reactions), which could impact nutritional anti‐inflammatory tactics and bone protection measures (such as adequate protein, calcium, and vitamin D). A preliminary attempt at targeted methods was made by an investigation that explicitly examined methyl‐donor intake (folic acid and vitamin B12) for women with SLE based on their nutritional state (da Mota et al. 2024). Yet there are not many explicit sex‐stratified diet RCTs in SLE. Individuals with SLE have been shown to exhibit age‐specific metabolomic characteristics, with notable trends such as lower levels of amino acids in younger individuals, higher levels of very‐low‐density lipoproteins in middle‐aged people, and higher levels of low‐density lipoproteins in older persons.

Although studies on nutrition have acknowledged the relevance of genetic variability in dietary supplement results, there are still few specific implications for SLE. The breakdown of essential nutrients for managing SLE, including folic acid, vitamin D, and omega‐3 fatty acids, is influenced by genetic variables. For example, inconsistency in vitamin D‐related behaviors may be partially explained by VDR polymorphisms (e.g., ApaI, BsmI) that have been linked to SLE vulnerability in meta‐analyses. This suggests a treat‐to‐level strategy (monitor 25(OH)D and titrate) as opposed to a preset dose (Yang et al. 2022).

There is still little data concerning individualized nutrition for SLE. To ascertain whether or not nutritional interventions tailored by race, gender, age, or family history offer benefits over broad advice, strong, segregated research studies are required. The development of customized dietary strategies which tackle unique biological characteristics and improve the health of patients is contingent on the integration of epigenetic, genetic, metabolomic, and microbiome‐related information with comprehensive clinical phenotyping.

## 5 Real World Limitations

Even though studies show that a thorough evaluation of dietary intake should be part of managing SLE and that a diet regimen may be an intriguing approach that offers preventive benefits without the adverse outcomes of traditional drugs, significant obstacles stand in the way of the successful integration of dietary intake into clinical care. Nutrition education for physicians is still scarce; surveys and reviews frequently reveal insufficient course duration and minimal guidance confidence, which turns into insufficient, short lifestyle exchanges in the clinic. Limited counseling reimbursement, divergent goals, a shortage of hands‐on training, and a paucity of availability are some of the identified obstacles.

Many worldwide healthcare standards still do not address medical nutrition therapy (MNT) for SLE, demonstrating a basic discrepancy between clinical recommendations and research findings. Although registered dietitian nutritionists (RDNs) are recognized by the American College of Rheumatology as members of the group of healthcare professionals assigned to treat individuals who have rheumatic ailments, this endorsement has not resulted in their widespread inclusion. Incorporating RDNs into interdisciplinary therapy groups would give professionals specialized knowledge that aids people in following dietary recommendations, enhancing health and lowering the likelihood of comorbidities.

Socioeconomic considerations have a substantial impact on the response of patients to dietary therapy. Higher body mass index (BMI) has been linked in studies to poorer socioeconomic position, lower educational attainment, higher levels of illness activity, greater corticosteroid amounts, and less physical activity. Complex obstacles to achieving nutritional modifications are created by these interrelated issues. Communication breakdowns between patients and providers have a big influence on compliance. According to studies, patients frequently fail to comprehend their illness and the drugs they are taking. One of the biggest obstacles is that they might not believe that their health will get worse if they do not get therapy. Individuals may not comprehend the link between food and illness management when receiving nutritional guidance, which is another area where these interpersonal problems arise.

While avoiding ultra‐processed foods, added sugars, and saturated fats, an anti‐inflammatory diet high in fruits, vegetables, whole grains, legumes, nuts, seeds, and omega‐3‐rich fish should be promoted. Those on corticosteroids or with little sun contact should pay special attention to getting enough calcium (1000–1200 mg/day) and vitamin D through periodic assessment. In LN, renal function is protected by reduced sodium intake (< 2 g/day) and customized protein and electrolyte modifications. In addition to maintaining good intestinal health with items high in fiber and probiotics, keeping an appropriate body weight through mindful eating and activity tailored to joint and fatigue state will contribute to improved overall results.

## 6 Clinical Translation Roadmap

The initial step in implementing individualized diets in SLE is to evaluate nutrient risk using proven instruments like the Mini Nutritional Assessment (MSA) or the Malnutrition Universal Screening Tool (MUST), in addition to culturally relevant nutritional evaluations. This assessment is combined with biological profiling, which includes metabolomic indications like SCFAs, particularly butyrate, which promotes Treg‐mediated immunological control, and blood biomarkers involving 25 (OH)D, folate, B12, and omega‐3 indexes. Such information, along with medical phenotype, disease progression, concurrent medical conditions, and multi‐omics perspectives, leads to individualized nutrition regimens segregated by race, gender, age, and genetic makeup. Adaptive nutrient focus change is made possible by ongoing biomarker surveillance, which replaces stagnant suggestions with adaptable, treat‐to‐biomarker management.

## 7 Conclusion

In SLE, dietary modification provides a viable supplement to traditional treatment by affecting certain proinflammatory and physiological processes. Certain fatty acids, micronutrients, polyphenols, and microbiota‐focused approaches have been shown to have beneficial effects on cardiovascular wellness and immunological modulation. The lack of rigorous trials, uneven biomarker usage, and varied food regimens, however, restrict clinical recognition. Personalized nutritional alteration, validated measurement of results, and accurate nutrient profiling will all be essential components of well‐powered, long‐term research that will drive advancements. Nutrition may be transformed from an auxiliary strategy to an organized, evidence‐based part of managing SLE by incorporating such methods into holistic treatment, which would enhance results while reducing the need for medications.

## Author Contributions

**Xiaowei Zhang:** software (equal), writing – original draft (equal), writing – review and editing (equal). **Guoliang Fan:** supervision (equal), writing – original draft (equal).

## Ethics Statement

The authors have nothing to report.

## Consent

All authors are willing for the publication of this manuscript.

## Conflicts of Interest

The authors declare no conflicts of interest.

## Data Availability

Even though adequate data have been given in the form of tables and figures, all authors declare that if more data is required, then the data will be provided on a request basis.
